# Xanthogranulomatous Pyelonephritis Associated with Hepatic Dysfunction in Pregnancy

**DOI:** 10.1155/2015/936262

**Published:** 2015-05-21

**Authors:** L. Ferreira, C. Oliveira, C. Cruz, A. Pacheco

**Affiliations:** Maternal-Foetal Medicine Unit, Faro Hospital, Faro, Portugal

## Abstract

Xanthogranulomatous pyelonephritis is a rare disease characterised by the replacement of normal renal parenchyma by foamy macrophages. The only treatment for this type of pyelonephritis is of a surgical nature with partial or total nephrectomy. The occurrence of xanthogranulomatous pyelonephritis during pregnancy is a rare event (with only 6 cases described in the literature). We report a case of xanthogranulomatous pyelonephritis in a 32-week pregnant woman associated with hepatic dysfunction.

## 1. Introduction

Xanthogranulomatous pyelonephritis (XGP) was first described in 1916 by Schlagenhauer [1] and it is still a rare and serious condition today. This type of pyelonephritis is usually a unilateral and diffuse process, characterised by the replacement of normal renal parenchyma with foamy macrophages [2]. The exact etiology of XGP is still unknown; however, it is associated with long-term renal obstruction with concomitant infection and an inadequate inflammatory response to this process [3].

The disease is typically diagnosed in women in their 50s and 60s. Clinical features are identical to other forms of pyelonephritis except for the weight loss and rundown appearance that are usually present [4].

As far as treatment is concerned, in most cases, a nephrectomy is necessary. After surgery, long-term follow-up is necessary with annual imaging of the contralateral kidney and aggressive treatment of urinary infections [5].

To our knowledge, there are six cases of XGP during pregnancy reported. Here we describe a case of XGP diagnosed in a pregnant woman.

## 2. Case Report

A 25-year-old woman, primigravida, first visited our hospital at 32 weeks due to a fever and right flank pain. These symptoms had begun 3 days prior to her visit. She reported several episodes of urinary-tract infections (UTI) in the past, associated with urolithiasis. Her past surgical history was irrelevant to this case. She had no significant family history.

Upon physical examination, the patient presented hyperthermia (38.4°C), tachycardia (110 beats per minute), and blood pressure of 110/85 mmHg. During abdominal examination, there was suprapubic tenderness without rebound pain and right flank tenderness at percussion. Gynaecological examination was normal. Obstetric ultrasound revealed a foetus growing in the 50th percentile with normal corporal movements and amniotic fluid and a cervical length of 35 mm. A cardiotocography was performed and did not register contractility activity.

Analytical evaluation revealed an elevated white blood cell count (15 × 10^3^/*μ*L) with 71% neutrophils and 18% lymphocytes, with normal haemoglobin (Hb 12 g/dL) and platelet count (400 × 10^9^/L), an elevated protein C reaction (92 mg/dL), and mild elevation of liver enzymes (aspartate aminotransferase of 48 UI/L, alanine aminotransferase of 107 UI/L, and alkaline phosphatase of 346 UI/L). Lactate dehydrogenase was normal (105 IU/L). Urine tested positive for leucocytes and proteinuria but negative for red blood cells.

The patient was admitted to our maternal-foetal unit with the diagnosis of acute pyelonephritis and treatment was started empirically with ampicillin and gentamicin after urine and blood culture were performed.

Despite treatment, the fever persisted and the patient's condition did not improve. An increase in protein C reaction and in liver enzyme levels (aspartate aminotransferase of 92 UI/L, alanine aminotransferase of 147 UI/L, total bilirubin of 1.8 mg/dL with direct bilirubin of 0.9 mg/dL, and alkaline phosphatase of 354 UI/L) was observed. Other causes of hepatic dysfunction in pregnancy as preeclampsia and hemolysis and elevated liver enzymes and low platelets (HELLP) were considered but excluded due to absence of hypertension and normal platelet values.

Urine culture was positive for* Proteus mirabilis* and a renal ultrasonography, performed upon admission, was suggestive of pyonephrosis: the right kidney of 181 mm had a thinned parenchyma and a dilated collecting system with multiple hyperechogenic foci.

The patient was then referred to a urologist and treatment plan was reevaluated, which led to the placement of a double-J-stent in the affected kidney. Prior to surgery, betamethasone was administered to promote foetal lung maturity. The stent was successfully placed via cystoscopy under anaesthesia and atosiban infusion. Clinical and analytical improvement was observed 24 hours after stent placement. The patient was discharged at day 19.

The rest of the antenatal period was uneventful; nonetheless an ambulatory monitoring of her liver and kidney function as well as of foetal wellbeing was repeated every two weeks.

At 37 weeks, after spontaneous onset of labour, the patient gave birth to a male newborn of 2560 g through vaginal delivery.

**Figure 1:**
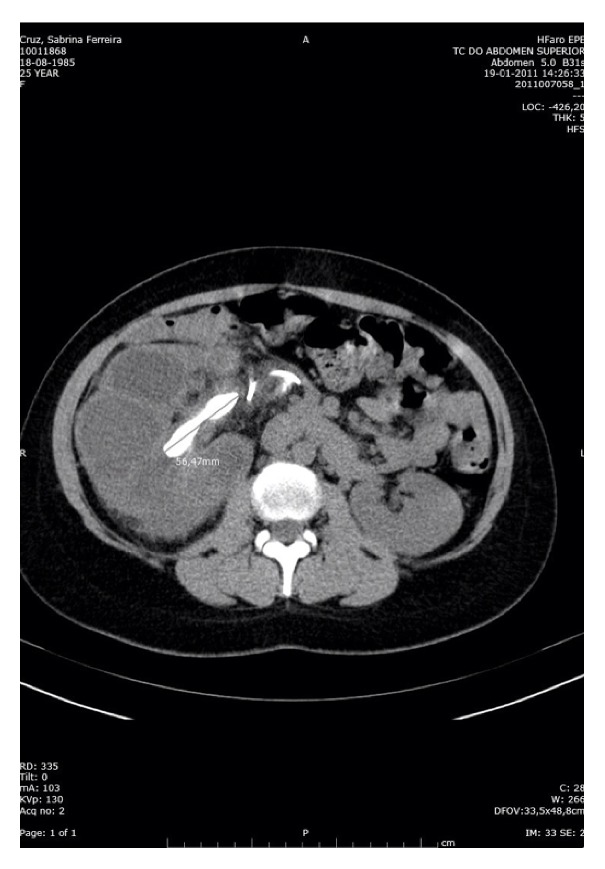
Abdominal pelvic CT showed an enlarged right kidney of 164 mm.

**Figure 2:**
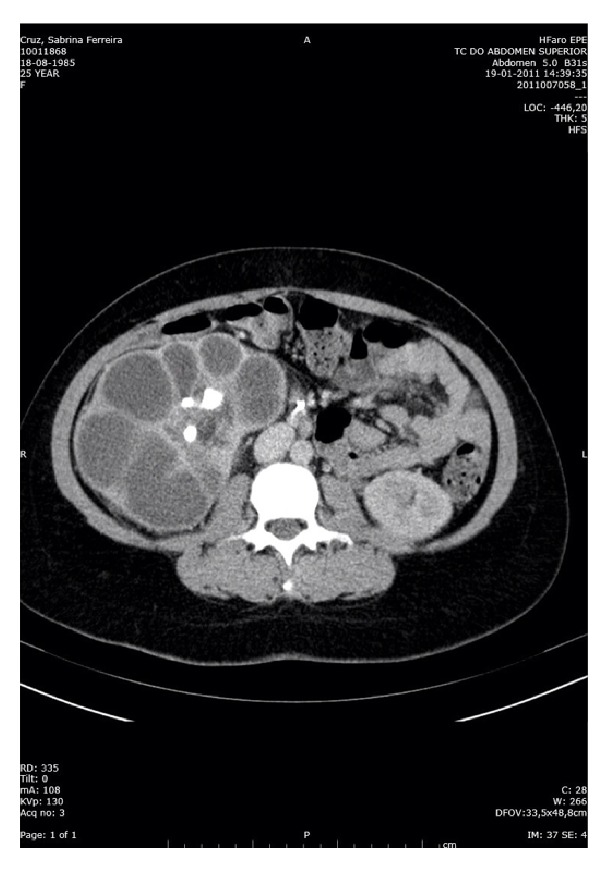
Abdominal pelvic CT: presence of a dilated collecting system with the presence of a coraliform calculus of 55 mm in the renal pelvis.

Abdominal pelvic CT performed 1 month after birth showed an enlarged right kidney of 164 mm (Figure 1) with a dilated collecting system with the presence of a coraliform calculus of 55 mm in the renal pelvis (Figure 2).

The double-J-stent was not removed and the patient was submitted to a unilateral nephrectomy 6 months after delivery.

The pathological examination was compatible with diffuse xanthogranulomatous pyelonephritis.

## 3. Discussion

Xanthogranulomatous pyelonephritis is a rare type of pyelonephritis, found in 0.6 to 1.4% of cases of renal inflammation [4, 12]. This slowly progressive renal infection is even rarer in pregnancy; only 6 cases of XGP in pregnancy have been reported [6–11].

The low incidence of this disease and three features make this a distinctive case.

Firstly, its association with pregnancy: the etiology of this disease is still unclear; however there are several factors associated with its appearance, such as urinary-tract obstruction and altered immunological responses. In this case, pregnancy and repeated episodes of urolithiasis in the past may have led to this type of pyelonephritis.

In general, hormonal and mechanical changes in pregnancy increase the risk of urinary stasis and vesicoureteral reflux predisposing some women to upper urinary-tract infections (UTIs) and acute pyelonephritis. Indeed, UTIs are among the most common bacterial infections during pregnancy [13].

In five cases published of XGP during pregnancy, a renal calculus was present, increasing urinary stasis. As in the case presented by the authors, the main symptom that led to the diagnosis was fever and pain. Contrary to our case, the five cases were diagnosed before 16 weeks and only one occurred during the third trimester (Table 1).

Secondly, the presence of hepatic dysfunction: former reports focused on systemic manifestations of XGP, one consisting of liver manifestations [11, 14, 15]. Indeed, in 50% of patients with XGP, an abnormal liver enzymes level is observed [16]. More recent reports describe cases of XGP with extensive infiltration of the liver parenchyma. In relation to pregnancy, Sworn and Jones [11] reported a case of hepatic dysfunction postpartum due to XGP. In that specific case, there was no normalization of the liver enzymes value after surgical treatment.

In the case presented by the authors, the inflammatory changes responsible for the chronic infection were probably responsible for the elevation of the liver enzymes and, as reported in published cases, the surgical treatment with nephrectomy led to the normalization of the liver function.

Other maternal and foetal complications, such as septic shock, renal dysfunction, pulmonary injury, and premature delivery, are associated with pyelonephritis. Elective abortion followed by premature delivery was the complication most frequently described in the 6 pregnant women with XGP.

The third point relates to diagnosis and type of treatment. Ultrasonography images may suggest the presence of XGP [17]; however, computed tomography (CT scan) and magnetic resonance imaging (MRI) are more sensitive. MRI has high sensitivity and specificity to identify fat as hyperintense signal on T1 and T2 weighted images with the advantage in pregnancy of not using contrast material or ionized radiation [18]. In the case presented by Badar et al. [7], the MRI findings led to the diagnosis of renal replacement lipomatosis with coexistent XP. In our case, an MRI was not performed during pregnancy due to unavailability; instead a CT scan was performed prior to surgery to evaluate the extension of the inflammatory process.

Indeed computed tomography is the technique of choice to determine the extent of parenchyma and the presence of complications [19]. CT imaging grades XGP into 3 stages: stage I (nephric) is a localized disease confined to the renal parenchyma; stage II (perinephric) lesions involve perinephric fat; and stage III (paranephric) lesions extend beyond Gerota's fascia into the retroperitoneum [20]. In the case presented, the CT scan performed was suggestive of a stage I XGP.

Concerning management, the only effective treatment in diffuse XGP is a surgical one, with partial or total nephrectomy.

Our case diverges from that of Figueroa et al. [8] as in this case the placement of a percutaneous nephrostomy was not effective, which led to a nephrectomy during pregnancy. In the case presented, the placement of a double-J-stent was effective and permitted postponing surgery until after the delivery.

This case highlights the importance of considering differential diagnosis, particularly when inflammatory parameters are present. The diagnosis of XGP should be considered in the presence of a complicated pyelonephritis associated with hepatic lesion.

## Figures and Tables

**Table 1:** Comparison of published cases.

	Age (years)	Gestational age (weeks)	Pyrexia	Pain	Flank mass	Calculi	Urine culture	Pregnancy evolution	Nephrectomy
Bianchi and Franzolin [6]	27	16	+	+	−	+	*Staphylococcus Proteus *	Elective abortion	Unilateral

Badar et al. [7]	30	12	−	−	+	+	*Staphylococcus *	Uneventful	Unilateral (during pregnancy)

Figueroa et al. [8]	24	12	+	+	−	+	*Proteus mirabilis *	Uneventful	Unilateral nephrectomy

Loffroy et al. [9]	37	3rd trimester	+	+	−	—	—	Elective preterm birth	Nephrectomy postpartum

Ballesteros Sampol et al. [10]	—	14	+	+	−	−	*Escherichia coli *	Elective abortion	Unilateral nephrectomy

Sworn and Jones [11]	29	Postpartum	+	−	+	+	*Escherichia coli Klebsiella *	—	Nephrectomy postpartum

Ferreira et al. (current case)	25	32	+	+	−	+	*Proteus mirabilis *	Uneventful	Nephrectomy postpartum
